# Long-term dyspnea, regional ventilation distribution and peripheral lung function in COVID-19 survivors: a 1 year follow up study

**DOI:** 10.1186/s12890-022-02214-5

**Published:** 2022-11-09

**Authors:** Gaetano Scaramuzzo, Luca Ronzoni, Gianluca Campo, Paolo Priani, Chiara Arena, Riccardo La Rosa, Cecilia Turrini, Carlo Alberto Volta, Alberto Papi, Savino Spadaro, Marco Contoli

**Affiliations:** 1 Department of Translational Medicine, University of Ferrara, Via Aldo Moro 8, 44121 Ferrara, Italy; 2 Anesthesia and Intensive Care Medicine, Azienda Ospedaliero Universitaria Di Ferrara, Via Aldo Moro 8, 44124 Cona, FE Italy; 3 Respiratory Medicine, IRCCS Reggio Emilia, Arcispedale Santa Maria Nuova, Reggio Emilia, Italy; 4 Cardiology Unit, Azienda Ospedaliero Universitaria Di Ferrara, Via Aldo Moro 8, 44124 Cona, FE Italy; 5 Respiratory Medicine, Azienda Ospedaliero Universitaria Di Ferrara, Via Aldo Moro 8, 44124 Cona, FE Italy

**Keywords:** COVID-19, Post COVID-19, Electrical impedance tomography, Spirometry, Pulmonary function test, Dyspnea

## Abstract

**Background:**

Dyspnea is common after COVID-19 pneumonia and can be characterized by a defective CO_2_ diffusion (DLCO) despite normal pulmonary function tests (PFT). Nevertheless, DLCO impairment tends to normalize at 1 year, with no dyspnea regression. The altered regional distribution of ventilation and a dysfunction of the peripheral lung may characterize dyspnea at 1 year after COVID-19 pneumonia. We aimed at assessing the pattern of airway resistance and inflammation and the regional ventilation inhomogeneity in COVID-19 pneumonia survivors at 12-months after hospital discharge.

**Methods:**

We followed up at 1-year patients previously admitted to the respiratory units (intensive care or sub-intensive care unit) for COVID-19 acute respiratory failure at 1-year after hospital discharge. PFT (spirometry, DLCO), impulse oscillometry (IOS), measurements of the exhaled nitric oxide (FENO) and Electrical Impedance Tomography (EIT) were used to evaluate lung volumes, CO_2_ diffusion capacity, peripheral lung inflammation/resistances and the regional inhomogeneity of ventilation distribution. A full medical examination was conducted, and symptoms of new onset (not present before COVID-19) were recorded. Patients were therefore divided into two groups based on the presence/absence of dyspnea (defined as mMRC ≥1) compared to evaluate differences in the respiratory function derived parameters.

**Results:**

Sixty-seven patients were admitted between October and December 2020. Of them, 42/67 (63%) patients were discharged alive and 33 were evaluated during the follow up. Their mean age was 64 ± 11 years and 24/33 (73%) were males. Their maximum respiratory support was in 7/33 (21%) oxygen, in 4/33 (12%) HFNC, in 14/33 (42%) NIV/CPAP and in 8/33 (24%) invasive mechanical ventilation. During the clinical examination, 15/33 (45%) reported dyspnea. When comparing the two groups, no significant differences were found in PFT, in the peripheral airway inflammation (FENO) or mechanical properties (IOS). However, EIT showed a significantly higher regional inhomogeneity in patients with dyspnea both during resting breathing (0.98[0.96–1] vs 1.1[1–1.1], *p* = 0.012) and during forced expiration (0.96[0.94–1] vs 1 [0.98–1.1], *p* = 0.045).

**Conclusions:**

New onset dyspnea characterizes 45% of patients 1 year after COVID-19 pneumonia. In these patients, despite pulmonary function test may be normal, EIT shows a higher regional inhomogeneity both during quiet and forced breathing which may contribute to dyspnea.

**Clinical trial registration:**

Clinicaltrials.gov NCT04343053, registration date 13/04/2020.

**Supplementary Information:**

The online version contains supplementary material available at 10.1186/s12890-022-02214-5.

## Background

The post-COVID (or long COVID) syndrome (PCS) is defined as the persistence of symptoms after 12 weeks from a probable or confirmed SARS-CoV-2 infection and not explained by an alternative diagnosis [1, 2]. Dyspnea, anosmia/dysgeusia, fatigue and neurological disorders are the most commonly reported symptoms [2] and can affect more than 70% of patients after 1 year from hospital discharge [3]. Despite persistent dyspnea has been reported in more than half of patients affected by PCS [4] and may reduce health-related quality of life and functional status [5], its pathophysiology has not been totally understood by now.

Dyspnea can be described as “a subjective experience of breathing discomfort that consists of qualitatively distinct sensations that vary in intensity” [6]. Dyspnea can affect quality of life, exercise tolerance and mortality in various diseases and conditions. Despite being a symptom characterizing many different diseases, the underlying mechanisms of dyspnea are still poorly understood [7] and are probably complex and multifactorial [8].

New onset dyspnea after COVID-19 has been associated with an increase of peripheral airway resistances [9, 10] and/or a reduced diffusion lung capacity (DLCO) [11–13]. These phenomena may derive from the impairment of the peripheral lung (inhomogeneity of peripheral ventilation, airway/parenchyma damage, microvascular abnormalities, or a combination of these events) and even though many studies based on CT scan looked for anatomical abnormalities, the functionality of this lung region is still largely unexplored.

The properties of the peripheral lung may be assessed by different techniques. Specifically, measurement of fractional nitric oxide (NO) concentration in exhaled breath (FENO), impulse oscillometry (IOS) and Electrical Impedance Tomography (EIT) could be used to investigate airway inflammation, airway resistance and regional ventilation distribution respectively.

FENO is a quantitative, non invasive method [14, 15] that provides data on NO production, and thus can reflect airway inflammation [16], while impulse oscillometry (IOS) can be useful to test airway resistances [17, 18]. In addition, also Electrical impedance tomography (EIT) could be useful in this context. EIT is a non-invasive monitoring technique that can be used to assess the regional inhomogeneity, both during quiet and forced breathing [19]. Static and dynamic volumes may be indeed preserved in PCS patients with dyspnea [20], but their relative distribution may be altered.

Our hypothesis was that the peripheral lung function and/or the homogeneity of ventilation may be altered at 1 year from COVID-19 pneumonia and that this impairment may be related to the occurrence of dyspnea in PCS. To test these hypotheses, we assessed the pattern of airway resistance, airway inflammation and the regional ventilation inhomogeneity, along with classical pulmonary function tests (PFT) such as spirometry and DLCO, in COVID-19 pneumonia survivors at 12-months after hospital discharge. Moreover, we used IOS, FENO and EIT, to evaluate if peripheral lung dysfunction and/or ventilation inhomogeneity could be associated to new onset dyspnea related to the PCS.

## Methods

### Study population

This is a prospective cohort study of COVID-19 pneumonia survivors admitted to the respiratory and Intensive care units of the Arcispedale Sant’Anna Hospital (Ferrara, Italy) between October and December 2020. Inclusions criteria were: age > 18 years; confirmed SARS-CoV-2 infection; hospitalization for acute respiratory failure (defined as a PaO2/FiO2 (P/F) ratio ≤ 200 mmHg); need for invasive or noninvasive mechanical ventilation or only oxygen support. SARS-CoV-2 infection was confirmed by reverse-transcriptase-polymerase-chain-reaction assay (Liaison MDX, Diasorin, Saluggia, Italy) from a nasopharyngeal swab specimen.

At 12 months from hospital discharge, the survivors of the original cohort were contacted to set up a date for a complete respiratory evaluation, including a medical interview and respiratory function tests. The following exclusions criteria were verified before performing EIT, according to the current evidence [21]: 1) the presence of pacemaker/ICD, 2) pregnancy and 3) impossibility to position the EIT belt (e.g. for the presence of chest lesions).

The study was approved by the ethical committee of the Emilia Romagna (AVEC ethical committee) and informed consent was obtained by the patient or by the next of kin according to the approval of the local Ethics committee. Clinicaltrials.gov: NCT04343053; registration date 13/04/2020.

### Data acquisition protocol and definitions

During the follow up visit, all patients underwent a complete sequential evaluation of respiratory function, including the following exams: 1) FeNO, 2) spirometry, 3) DLCO, 4) oscillometry and 5) Electrical impedance tomography. Thereafter, the recent patients’ history, focusing on symptoms of new onset experienced during the last year and not present prior to SARS-CoV-2 infection, was collected. Finally, we evaluated and recorded the severity of dyspnea using the modified Medical Research Council (mMRC) scale [22]. Dyspnea associated to the PCS (reported from now simply as “dyspnea”) was defined as dyspnea (mMRC≥1) that started after the SARS-CoV-2 infection, was still present after at least 12 weeks and was not explained by an alternative diagnosis [1]. Data about lung imaging and/or cardiological check-up done after hospital dismission and before follow up visit were also acquired.

### Respiratory function assessment: FENO, Spirometry, DLCO and oscillometry

The exhaled fraction of NO (FENO) was measured with an electrochemical analyzer (FeNO+; Medisoft, Sorinnes, Belgium), according to the current guidelines [15]. The patients were instructed to exhale at multiple flows (50, 100 and 150 ml/s) starting from a flow of 50 ml/s and increasing up to 150 ml/s. The alveolar NO concentration (Calv, Jaw) was therefore automatic calculated by the system based on mathematical model [23].

Spirometry was performed after FeNO, according to ATS/ERS standards [24]. To evaluate large airways obstruction, we recorded the percentage of predicted FEV1, FEV1/FVC, FVC. We also evaluated the diffusing capacity for carbon monoxide (DLCO) and the diffusing capacity for carbon monoxide/alveolar volume, as previously described [25]. In addition, impulse oscillometry [26] (IOS, Vmax and Jaeger Master Screen-IOS; Carefusion Technologies, San Diego, CA, USA) was used to assess resistances and reactance of the respiratory system. The following parameters were calculated using IOS derived data: the difference between resistance at 5 Hz and resistance at 20 Hz (i.e. R5–R20), the area of reactance (AX) and the respiratory system reactance (X5).

### Lung regional distribution imaging: electrical impedance tomography

Patients were monitored using a 16 electrodes EIT system. The system was composed of a belt, chosen according to the size of the chest-wall and placed around the 4th–5th intercostal space and of a dedicated monitor (PulmoVista® 500, Dräger Medical, Lübeck, Germany). EIT recording was performed during spirometry, as previously described [19, 27].

After image acquisition, the following EIT images were reconstructed: end expiration (EE_EIT_), end inspiration (EI_EIT_) during resting breathing, at the point of maximal inspiration during forced inspiration (FI_EIT_) and 1 second after the start of forced expiration (FEV1_EIT_).

The tidal volume image (TV_EIT_) was obtained as EI_EIT_-EE_EIT_. The volume expired at FEV1 (∆FEV1_EIT_) was derived by subtracting the FEV1_EIT_ to FI_EIT_. For both TV_EIT_ and ∆FEV1_EIT,_ the coefficient of variation (CV) and the global inhomogeneity index (GI) were calculated as previously described [19, 28]. All images were acquired with the patients sitting in a relaxed position (90°).

### Statistical analysis

This is a longitudinal, prospective, follow-up, observational study. At the time of study design, no study was available for a formal sample size calculation. Therefore, we aimed at enrolling the higher number of patients available from the original cohort. Continuous data are presented as median and first-to-third interquartile range (IQR) or mean ± SD according to the results of the normality tests assumption, while categorical variables as counts (percentage). The normality of data distribution was tested using the Shapiro-Wilks normality test and data report and test selection was appropriately based on its results. Participants were categorized into two groups (symptomatic/asymptomatic for respiratory symptoms) according to the mMRC scale score during the follow up visit and defined as symptomatic if mMRC was ≥1 [29].

Comparison between independent groups was performed using the T-test, Mann-Whitney U test or Chi-square test, depending on the data type and distribution. All *P*-values refer to two-tailed tests of significance, and *p* < 0.05 was deemed as the statistically significant. Data were analyzed using SPSS Statistics 26 (IBM SPSS Statistics for Windows, Version 26.0. Armonk, NY: IBM Corp.) and GraphPad Prism 8.4.3 (GraphPad software, Northside Dr. Suite 560 San Diego).

## Results

**Fig. 1:**
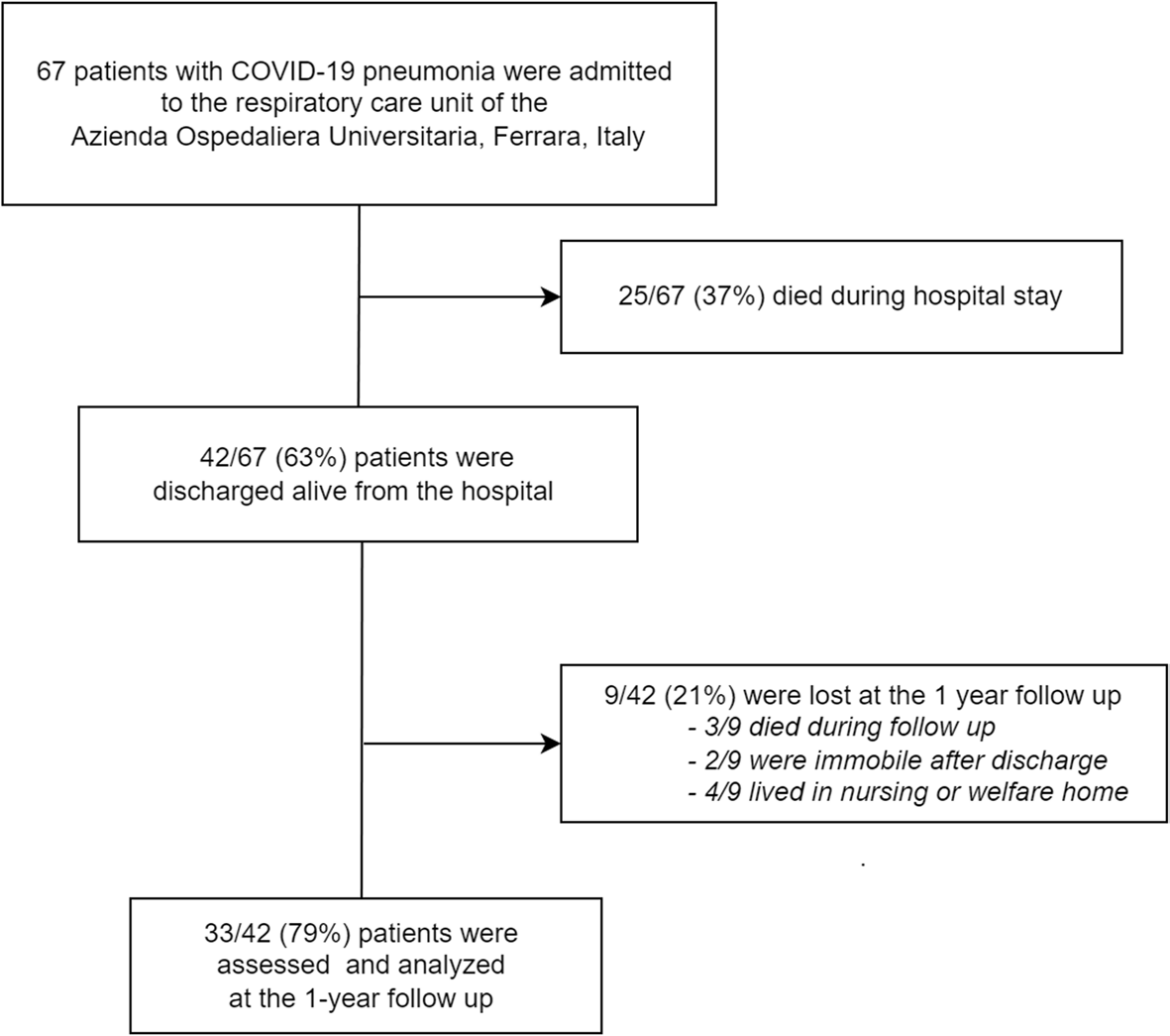
Flow chart of the study

Sixty-seven patients with COVID-19 pneumonia were admitted to the respiratory and Intensive care units of the Azienda Ospedaliera Universitaria of Ferrara (Ferrara, Italy). Of them, 25/67 (37%) died during hospital stay while 42/67 (63%) patients were discharged alive. Nine patients (9/42, 21%) were lost during the follow up (3/9 died, 2/9 were immobile after discharge and 4/9 were living in nursing or welfare home). All the remaining 33 patients were evaluated during the 1-year follow up visit (Fig. 1). The mean time of assessment was 380 ± 48 days after hospital discharge and the characteristics of the study population are reported in Table 1. Sixteen patients underwent lung imaging before the follow up visit while 7 patients underwent a complete cardiological check-up (Supplemental table 1).

**Table 1:** Demographic data of the population

	No Dyspnea (*n* = 18)	Dyspnea (*n* = 15)	Overall population (*n* = 33)	*P* value
Age (years)	62 ± 11	66 ± 11	64 ± 11	0.35
Male: Females ratio (N)	12:6	12:3	24:9	0.4
Height (cm)	172 ± 6	174 ± 10	173 ± 8	0.54
Weight (kg)	84 ± 12	88 ± 13	86 ± 13	0.29
BMI (kg/cm^2^)	28 ± 2.8	29 ± 3.9	28.5 ± 3.3	0.35
Smokers				0.14
*No, n (%)*	*14(77.8%)*	*8 (53.3%)*	*22/33 (66.7%)*	
*Current /Former smokers; n(%)*	*4 (22.2%)*	*7 (46.7%)*	*11/33 (33.3%)*	
COPD/Asthma (No. n(%))	17 (94.4%)	13 (86.7%)	30 (90.9%)	0.53
Admission to intensive care unit (yes)	13 (72.2%)	8 (53.3%)	21 (64%)	0.26
Days of hospital stay (days)	25[14–59]	25[11–40]	24 [13–43]	0.88
Days of ICU stay (days)	13[0–18]	3[0–16]	7[0–18]	0.18
Days of mechanical ventilation (days)	8.7 ± 16	1.5 ± 4.5	5 ± 12	0.09
Highest respiratory support during hospital stay (yes)				0.34
*Nasal cannula*	*2 (11%)*	*5 (33%)*	*7 (21%)*	
*HFNO*	*2 (11%)*	*2 (13%)*	*4 (12%)*	
*NIV*	*8 (44%)*	*6 (40%)*	*14 (42%)*	
*Endotracheal intubation*	*6 (33%)*	*2 (13%)*	*8 (24%)*	

*BMI* body mass index, *COPD* chronic obstructive pulmonary disease, *ICU* intensive care unit, *HFNO* high flow nasal oxygen, *NIV* non invasive ventilation

### Dyspnea at the 1-year follow up

New onset dyspnea was reported by 15/33 (45%) patients (Table 2). Of them, 9/15 reported an mMRC score = 1; 4/15 an mMRC score = 2 and 2/15 and mMRC score = 3. No differences were found among the two groups in the demographic characteristics and medical history. We found a higher but not statistically significant prevalence of patients that underwent mechanical ventilation (NIV/CPAP or invasive ventilation) in the group with no respiratory symptoms (14/18, 78%) as compared to patients with dyspnea (8/15, 46%, *p* = 0.14).

**Table 2:** Clinical data during medical consultation

	No Dyspnea (*n* = 18)	Dyspnea (*n* = 15)	Overall population (*n* = 33)	*P* value
Heart rate (bpm)	77 ± 12	77 ± 10	77 ± 11	0.94
SpO_2_ (%)	97 ± 1.5	97 ± 1.4	97 ± 1.5	0.32
Chest signs (yes)	7 (39%)	9 (60%)	15/33 (45%)	0.22
Respiratory symptoms (yes)	0	15 (100%)	15/33 (45%)	< 0.001
Other symptoms (yes)	10 (56%)	1 (7%)	11/33 (33%)	< 0.001
mMRC	0[0–0]	1[1–2]	0 [0–1]	< 0.001

*SpO*_*2*_ peripheral oxygen saturation, *mMRC* modified Medical Research Council (mMRC) scale

### Spirometry and DLCO

Respiratory function parameters derived from spirometry and DLCO evaluations are reported in Table 3. Three patients (9%) showed an FEV1/FVC < 0.70 and 6 patients (18%) had an FVC less than 80%, but no differences were found in lung function parameters when comparing symptomatic and non-symptomatic patients (Table 3). Overall, almost half of the patients (16/33, 49%) had a reduction in DLCO, with no significant difference between the two groups (*p* = 0.41).

**Table 3:** Spirometry and DLCO in symptomatic and non-symptomatic patients

	No Dyspnea (*n* = 18)	Dyspnea (*n* = 15)	Overall population (*n* = 33)	*P* value
FEV1 (liters)	2.9 ± 0.75	3 ± 0.8	3 ± 0.76	0.96
FEV1 (%)	100 ± 19.5	99.8 ± 19.7	100 ± 19	0.95
FVC (liters)	3.6 ± 0.93	3.6 ± 1.09	3.6 ± 0.9	0.99
FVC (%)	97 ± 17	98 ± 20	97 ± 18.6	0.95
FEV1/FVC (%)	103 ± 11.2	106.3 ± 6.3	105 ± 9.2	0.39
DLCO (mL/mmHg/min)	78.4 ± 16.7	83 ± 15.7	81 ± 16	0.41
DLCO/VA (mL/mmHg/min/L)	98.5 ± 16	104.9 ± 17	101 ± 16.4	0.27

*FEV1* Forced Expiratory Volume in the 1st second, *FVC* forced vital capacity, *DLCO* diffusing capacity for carbon monoxide, *VA* alveolar volume

### FeNO and impulse Oscillometry

Multiple-flow FeNO and IOS results are shown in Tables 4 and 5. Higher value of FeNO_150_ values were found in patients with dyspnea, but the difference did not reach statistical significance (*p* = 0.18). Moreover, no significant differences were found in terms of FeNO_50_, FeNO_100_, Calv and Jaw among the two groups (Tables 4 and 5). The evaluation of small airways showed no difference between the two groups as expressed by R5- R20 greater than 0.07 kPa/l/s (*p* = 0.99), AX (*p* = 0.63) and X5 (*p* = 0.76). A value of R5-R20 > 0.07 kPa/l/s was found in 5 patients without dyspnea (28%) and in 5 patients with dyspnea (33%, *p* = 0.99).

**Table 4:** Comparison of FENO data in patients with and without dyspnea

	No Dyspnea (*n* = 18)	Dyspnea (*n* = 15)	Overall population (*n* = 33)	*P* value
FENO_50_ (ppb)	15[9–21]	16[7–29]	16[9–23]	0.40
FENO_100_ (ppb)	12[9–15]	14.5[12–20]	13[9–16]	0.17
FENO_150_ (ppb)	8[6.7–11]	9[8.5–19]	9[7–12]	0.18
Calv	4.4 [2.8–10.5]	4.9[3.7–15.8]	4.5 [3.2–10.1]	0.6
Jaw	36 [16.5–54.3]	41 [22–104.8]	38 [19–64]	0.34

FENO50 = expired NO at 50 ml/sec. FENO100 = expired NO at 100 ml/sec. FENO150 = expired NO at 150 ml/sec

**Table 5:** Comparison of IOS data in patients with and without dyspnea

	No Dyspnea (*n* = 18)	Dyspnea (*n* = 15)	Overall population (*n* = 33)	*P* value
R5 (kpa)	0.45[0.36–0.72]	0.41[0.34–0.54]	0.45[0.36–0.58]	0.42
R20 (kpa)	0.43[0.33–0.48]	0.33[0.3–0.44]	0.38 [0.31–0.46]	0.21
R5-R20 (%)	11.5[1.3–16]	12[4.8–22.7]	11.7[4.6–19.9]	0.62
R5-R20 (kpa)	0.03[0.005–0.07]	0.04[0.02–0.11]	0.04[0.02–0.095]	0.40
R5-R20 > 0.07 (n, %)	5 (28%)	5 (33%)	10 (30%)	0.99
AX (kpa)	0.2[0.13–0.65]	0.22[0.16–0.57]	0.22[0.14–0.6]	0.66
X5 (kpa)	−0.095[−0.17-(−)0.067]	−0.1[−0.18-(−)0.08]	−0.1[−0.17-(−)0.07]	0.81

R5 = total respiratory system resistance, measured at 5 Hz. R20 = total respiratory system resistance, measured at 20 Hz. AX = Area of reactance; X5 = reactance at 5 Hz

### Regional distribution of ventilation

**Fig. 2:**
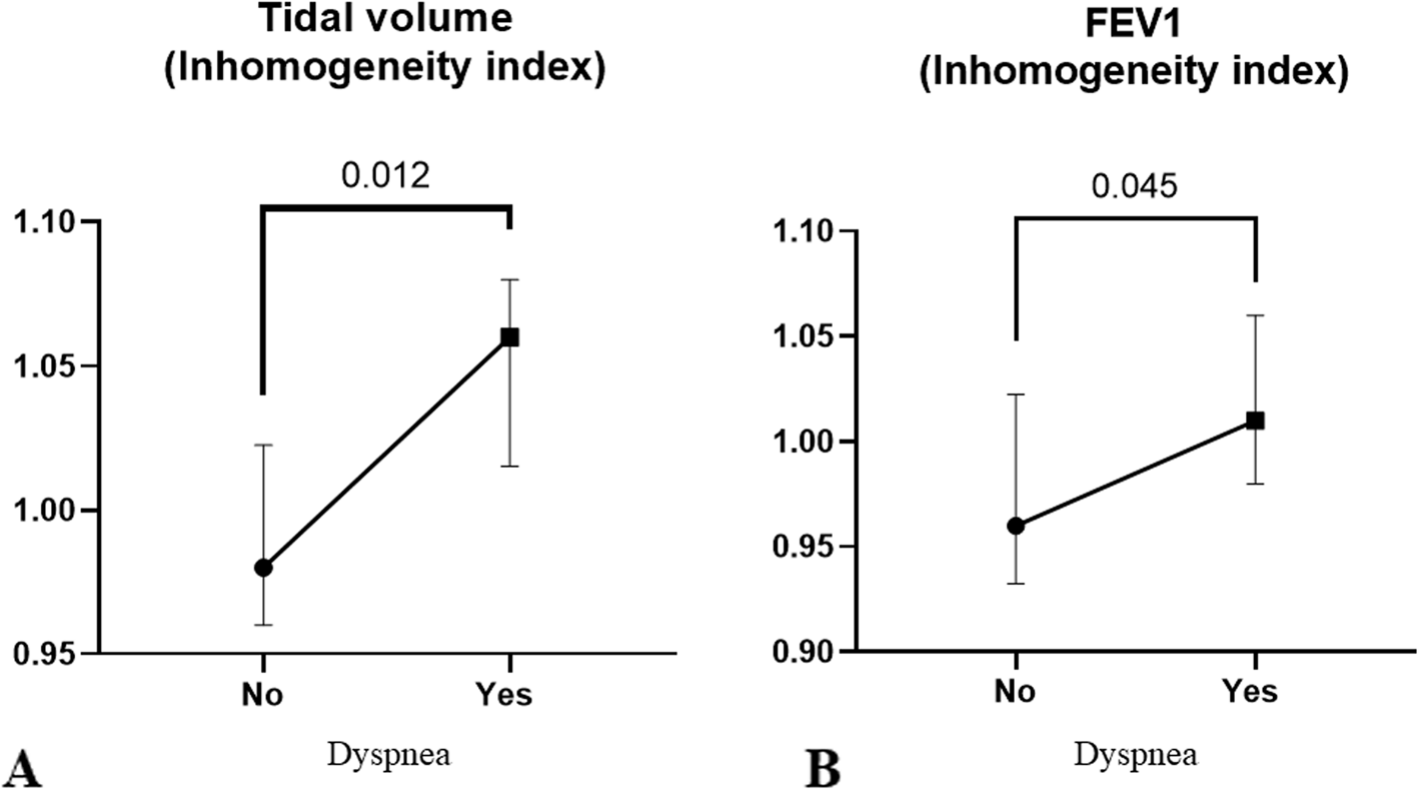
Inhomogeneity index derived from Electrical Impedance tomography (EIT) in patients with and without dyspnea during resting breathing (panel A, tidal volume) and forced expiratory maneuver (panel B)

**Fig. 3:**
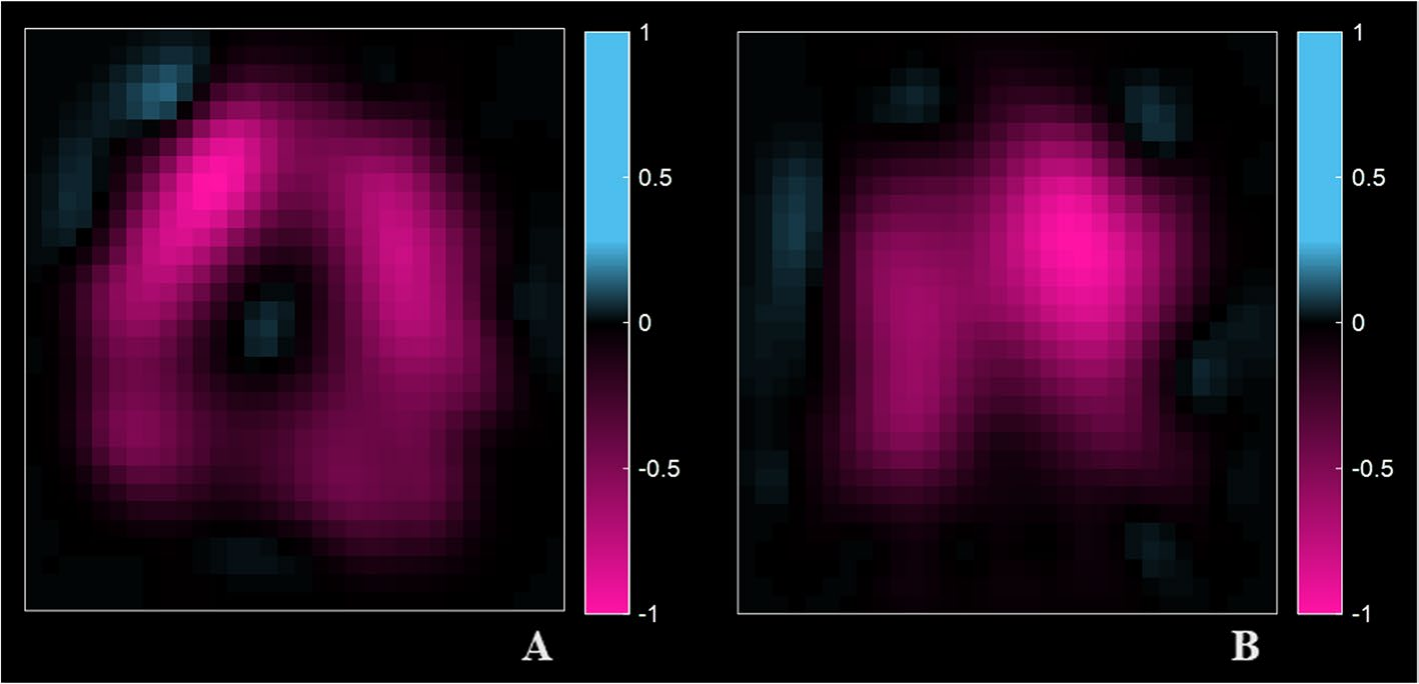
Example of EIT images during forced expiration maneuver (∆FEV1_EIT_) in two representative patients. Dynamic image of expired volume at 1 second during forced expiration (∆FEV1_EIT_) in two representative patients; 2**A**, asymptomatic patients (mMRC = 0); panel 2**B**: Symptomatic patient (mMRC = 2). Negative values represent the regional loss of volume (AU) in the 1st second after the start of forced expiration from vital capacity (FEV1)

When evaluating the inhomogeneity of ventilation using EIT derived CV, we did not find any significant difference between patients with and without dyspnea (Table 6). However, when comparing the global inhomogeneity (GI) indexes, a statistically significant difference was found between the two groups, with a higher level of inhomogeneity in patients with dyspnea, both during tidal breathing (GI of TV_EIT_ 0.98[0.96–1] vs 1.1[1–1.1], *p* = 0.012) and during forced expiration (GI of ∆FEV1_EIT_ 0.96 [0.94–1] vs 1 [0.98–1.1], *p* = 0.045, Table 6, Figs. 2 and 3).

**Table 6:** Electrical impedance tomography derived data

	No Dyspnea (*n* = 16)	Dyspnea (*n* = 13)	Overall population (*n* = 29)	*P* value
Tidal Volume (CV)	0.7[0.56–0.81]	0.73[0.69–0.85]	0.73[0.64–0.83]	0.29
∆FEV1 (CV)	0.6[0.5–0.69]	0.64[0.56–0.82]	0.6[0.52–0.75]	0.53
FVC (CV)	0.57[0.47–0.71]	0.64[0.54–0.78]	0.6[0.49–0.75]	0.25
Forced Inspiration (CV)	0.63[0.52–0.86]	0.64[0.58–0.76]	0.64[0.56–0.77]	0.71
Tidal volume (GI)	0.98[0.96–1]	1.1[1–1.1]	1[0.98–1.1]	***0.012***
∆FEV1 (GI)	0.96[0.94–1]	1[0.98–1.1]	0.98[0.95–1]	***0.045***
FVC (GI)	0.96[0.92–1]	0.99[0.98–1]	0.98[0.94–1]	0.08
Forced inspiration (GI)	1[0.96–1.1]	1[0.99–1.1]	1[0.97–1.1]	0.44

EIT data were missing for 4 patients

*CV* coefficient of variation, *GI* global inhomogeneity index, *FEV1* Forced Expiratory Volume in the 1st second, *FVC* forced vital capacity

## Discussion

Our study shows that, at 1 year from hospital discharge, the 45% of survivors admitted to the hospital for COVID-19 pneumonia experience dyspnea. In these patients, despite normal pulmonary function tests, the dynamic of regional ventilation is impaired and is characterized by a more inhomogeneous regional ventilation distribution in both resting and forced breathing.

The reduction of DLCO has been considered the main determinant of dyspnea in PCS [30]. Nevertheless, many recent evidence showed that DLCO tends to normalize or improve after 1 year, despite a non-neglectable number of patients in whom respiratory symptoms persist [12] and dyspnea remains, therefore, still unexplained. Also pulmonary function [31, 32] and cardiopulmonary exercise testing [33] do not show clear alterations in COVID-19 survivors experiencing PCS associated dyspnea.

We hereby evaluated two underexplored aspects in COVID-19 1-year survivors, which are 1) the peripheral lung inflammation and mechanical properties and 2) the regional distribution of ventilation, focusing on the homogeneity assessment.

Considering the peripheral lung function, the FENO and IOS analysis did not show any specific difference between the two groups, despite all the 4 patients with a FeNO_50_ > 25 ppb had dyspnea. This confirms what previously found in early PCS by Maniscalco et al. [34], who did not find any specific FENO increase in COVID-19 survivors as compared to healthy subjects. This was also noticed at 6-months from COVID-19 in a small cohort of patients, even though, in both cases, no assessment of dyspnea was performed [35]. The absence of small airway inflammation is also supported by not significantly increase of airways resistance, as assessed by impulse oscillometry. Previous studies, finding impaired oscillometry in the first 5 months after COVID-19 infection [9], also highlighted their significant improvement over time. In line with this and with the absence of high levels of FENO, airway inflammation may therefore characterize the first period of PCS, without justifying long-term (1-year) persistent dyspnea.

Interestingly, by acquiring EIT images, we were able to assess a new phenomenon which has not described before in the COVID-19 survivors and may go unrecognized using techniques who do not evaluate regional lung function. EIT was indeed able to reveal a higher level of inhomogeneity, during both resting breathing and forced expiration, despite normal respiratory function tests, in COVID-19 experiencing new onset dyspnea.

Electrical Impedance Tomography has been already extensively used in invasively ventilated [36–38], non-invasively ventilated [39] and spontaneously breathing patients [21] to evaluate lung inhomogeneity. The GI index has been previously associated to lung recruitment [28], weaning failure [40] and was used to assess patients with cystic fibrosis [41]. Moreover, GI was used to identify ventilation inhomogeneity in COPD patients in the phase of acute exacerbation [19].

Our findings may therefore unveil a new mechanism responsible for long COVID-19 dyspnea and potentially different from the altered diffusion and V/Q mismatch: a higher level of inhomogeneity may potentially alter the perception of effective ventilation and therefore cause dyspnea, despite global lung air content is maintained. This is also supported by previous evidences in obstructive [42] and neuromuscular diseases [43] patients that correlated lung ventilation inhomogeneity assessed by EIT with dyspnea. Our study confirmed therefore that COVID-19 survivors may be affected by dyspnea despite no alteration on the conventional PFT. Moreover, previous data were collected only few months after COVID-19 pneumonia [44] and no report evaluated regional ventilation distribution using EIT in COVID-19 survivors before.

Despite demonstrating that patients experiencing dyspnea had a higher level of ventilation inhomogeneity, the reason behind this phenomenon remains still unknown. One explanation may be the presence of anatomical abnormalities, such as residual fibrosis or parenchymal distortion, that could lead to an unregular intraparenchymal flow distribution. We found that, in dyspneic patients who underwent a radiological follow up, 3/7 showed a minimal residual fibrosis, especially in the peripheral lung. Further studies are needed to investigate if peripheral lung abnormalities may be associated to dyspnea and functional alterations, such as higher levels of intraparenchymal inhomogeneity.

Our study has some limitations. Firstly, the mMRC scale is subjective and therefore also determined by the patients psychosocial baseline characteristics. Secondly, in 4 patients it was not possible to perform EIT for technical problems with the EIT machine. Thirdly, we did not include a control group of non-COVID-19 patients. Further studies are needed to explore if this mechanism of heterogeneity of ventilation could explain dyspnea with no apparent cause also in non-COVID-19 patients. Fourth, EIT covers only a cross-section of the lung parenchyma, going generally from 5 to 10 cm and depending on the placement of the electrodes [45]. Despite this, EIT derived heterogeneity of ventilation has been previously found comparable to whole-lung MRI assessment in a translational study on rats [46], which is the current gold standard for ventilation heterogeneity assessment. Finally, our findings must be considered explorative since no sample size calculation was possible before the study design.

## Conclusions

New onset dyspnea characterizes 45% of patients 1 year after COVID-19 pneumonia. Patients with dyspnea at 1 year from COVID-19 pneumonia may have an altered and more inhomogeneous regional distribution of ventilation, despite no alteration in static volumes, resistances, and expired NO. Further studies are needed to assess potential biomechanical changes to ventilation in patients experiencing dyspnea and to evaluate if the inhomogeneity of ventilation distribution may be associated to the anatomical abnormalities using conventional radiology studies.

## Supplementary Information

**Additional file 1: Supplemental Table 1.** Patients with positive findings during radiological and cardiological follow up.

## Data Availability

The datasets used and/or analysed during the current study are available from the corresponding author on reasonable request.

## Abbreviations

∆FEV1_EIT_: Expired volume at 1 second after expiration EIT
AX: Area of reactance
BMI: Body mass index
Calv: Alveolar NO concentration
COPD: Chronic obstructive pulmonary disease
CPAP: Continuous positive airway pressure
DLCO/VA: Diffusing capacity for carbon monoxide on alveolar ventilation
DLCO: Diffusing capacity for carbon monoxide
EE_EIT_: End-expiratory EIT image
EIT: Electrical impedance tomography
FeNO: Franction of expired NO
FEV_EIT_: End-forced expiration image
FI_EIT_: Maximum inspiration EIT image
HFNC: High flow nasal cannula
ICU: Intensive care unit
IOS: Impulse oscillometry
NIV: Non invasive ventilation
PCS: Post-COVID-19 syndrome
R5–R20: Resistance at 5 Hz–resistance at 20 Hz
TI: Tidal image
TV: Tidal volume
TV_EIT_: Tidal volume EIT image
V/Q: Ventilation/perfusion
X5: Respiratory system reactance
