# Supplementary material for: Long-term dyspnea, regional ventilation distribution and peripheral lung function in COVID-19 survivors: a 1 year follow up study
Source: BMC Pulm Med. 2022 Nov 9;22:408. doi: 10.1186/s12890-022-02214-5 (PMC9643983; doi:10.1186/s12890-022-02214-5)
Supplement: Supplementary file 1 — Additional file 1: Supplemental Table 1. Patients with positive findings during radiological and cardiological follow up. [file 12890_2022_2214_MOESM1_ESM.docx]

**Supplemental table 1: Patients with positive findings during radiological and cardiological follow up**

|  | No Dyspnea | Dyspnea | Overall population |
| --- | --- | --- | --- |
| Positive radiological follow up (Chest X-ray or lung CT scan), nr. of patients | 1/9 | 3/7 | 4/16 |
| Positive Cardiological follow up (cardiological check-up), nr. of patients | 0/5 | 0/2 | 0/7 |

Number of patients with pathological findings in clinical exams (Lung CT scan, chest X ray or cardiological check-up) performed during the follow-up period. Of patients with positive findings at radiological follow up, 1 patient in the No Dyspnea group had a minimal peripheral fibrosis while, in the dyspnea group, 1 patient had minimal peripheral fibrosis and 2 showed disventilatory striae. None of the patients who underwent a cardiological follow up had pathological findings.
